# Perioperative use of serum creatinine and postoperative acute kidney injury: a single-centre, observational retrospective study to explore physicians’ perception and practice

**DOI:** 10.1186/s13741-021-00184-6

**Published:** 2021-05-25

**Authors:** Gianluca Villa, Silvia De Rosa, Caterina Scirè Calabrisotto, Alessandro Nerini, Thomas Saitta, Dario Degl’Innocenti, Laura Paparella, Vittorio Bocciero, Marco Allinovi, Angelo R. De Gaudio, Marlies Ostermann, Stefano Romagnoli

**Affiliations:** 1 Department of Health Sciences, Section of Anaesthesiology, Intensive Care and Pain Medicine, University of Florence, Viale Pieraccini 6, 50139 Florence, Italy; 2 Department of Anaesthesia and Intensive Care, Azienda Ospedaliero Universitaria Careggi, Florence, Italy; 3 Department of Anaesthesia and Intensive Care, San Bortolo Hospital, Vicenza, Italy; 4 Department of Biomedical Experimental and Clinical Sciences “Mario Serio”, University of Florence, Florence, Italy; 5 Department of Nephrology and Critical Care, Guy’s and St Thomas’ Hospital, King’s College London, London, UK

**Keywords:** Chronic kidney disease, Long-term kidney dysfunction, Glomerular filtration rate, Serum creatinine

## Abstract

**Background:**

Postoperative acute kidney injury (PO-AKI) is a leading cause of short- and long-term morbidity and mortality, as well as progression to chronic kidney disease (CKD). The aim of this study was to explore the physicians’ attitude toward the use of perioperative serum creatinine (sCr) for the identification of patients at risk for PO-AKI and long-term CKD. We also evaluated the incidence and risk factors associated with PO-AKI and renal function deterioration in patients undergoing major surgery for malignant disease.

**Methods:**

Adult oncological patients who underwent major abdominal surgery from November 2016 to February 2017 were considered for this single-centre, observational retrospective study. Routinely available sCr values were used to define AKI in the first three postoperative days. Long-term kidney dysfunction (LT-KDys) was defined as a reduction in the estimated glomerular filtration rate by more than 10 ml/min/m^2^ at 12 months postoperatively. A questionnaire was administered to 125 physicians caring for the enrolled patients to collect information on local attitudes regarding the use of sCr perioperatively and its relationship with PO-AKI.

**Results:**

A total of 423 patients were observed. sCr was not available in 59 patients (13.9%); the remaining 364 (86.1%) had at least one sCr value measured to allow for detection of postoperative kidney impairment. Among these, PO-AKI was diagnosed in 8.2% of cases. Of the 334 patients who had a sCr result available at 12-month follow-up, 56 (16.8%) developed LT-KDys. Data on long-term kidney function were not available for 21% of patients. Interestingly, 33 of 423 patients (7.8%) did not have a sCr result available in the immediate postoperative period or long term. All the physicians who participated in the survey (83 out of 125) recognised that postoperative assessment of sCr is required after major oncological abdominal surgery, particularly in those patients at high risk for PO-AKI and LT-KDys.

**Conclusion:**

PO-AKI after major surgery for malignant disease is common, but clinical practice of measuring sCr is variable. As a result, the exact incidence of PO-AKI and long-term renal prognosis are unclear, including in high-risk patients.

**Trial registration:**

ClinicalTrials.gov, NCT04341974.

## Background

Major surgery is a high-risk clinical condition complicated by acute kidney injury (AKI) (Calvert and Shaw 2012; Iyigun et al. 2019; O’Connor et al. 2016; Romagnoli et al. 2016). Besides cardiac surgery, major abdominal surgery is also associated with a high incidence of postoperative AKI (PO-AKI) (Romagnoli et al. 2016). Several studies have demonstrated that PO-AKI increases the risk of in-hospital and long-term mortality as well as progression toward chronic kidney disease (CKD) and end-stage renal disease (ESRD) (Palant et al. 2017).

Prevention and timely diagnosis of AKI are essential factors for improving patient outcomes (Cerda et al. 2018). Newly developed biomarkers, technologically advanced sniffers (electronic surveillance tools incorporated in a patient’s healthcare record to allow for timely diagnosis of AKI) or minimally invasive renal stress tests reported in the literature are all examples of the scientific community’s huge efforts to develop concepts and/or bed-side tools for earlier identification of patients susceptible to develop PO-AKI (Husain-Syed et al. 2018). However, the difficulties and costs associated with most of these newly developed technologies make their routine application in surgical patients very limited.

Currently, the identification of patients with high susceptibility to develop PO-AKI is mainly based on the recognition of risk factors for perioperative kidney impairment and on the changes in serum creatinine (sCr). Indeed, under several clinical conditions (e.g. high-risk procedures) and in some specific patients (e.g. elderly individuals or patients with pre-existing CKD), the renal functional reserve (RFR) might have already been impaired preoperatively (Hobson et al. 2017). Despite the well-known limitations (De Rosa et al. 2016), the preoperative value of sCr is currently used to examine patients’ baseline renal function. For this reason, the most recent clinical guidelines on diagnosis, grading, and management of AKI strongly suggest serial evaluation of sCr along with urinary output after high-risk conditions (e.g. major abdominal surgery), as well as long-term follow-up for those patients who experienced PO-AKI (Kellum et al. 2012).

In spite of these recommendations, PO-AKI might still be underdiagnosed, even in individuals preoperatively identified as high-risk patients, and long-term follow-up after AKI is usually not provided (Meersch et al. 2017). Within these premises, the primary aim of the present study is to describe the physician’s attitude toward the use of perioperative sCr for the identification of patients at risk for PO-AKI and long-term CKD. In particular, we will describe the perioperative approach adopted in a cohort of adult oncological patients undergoing major abdominal surgery at the Department of Anaesthesia of Careggi Hospital (i.e. a large tertiary care teaching hospital in Florence, Italy), and we will explore the physicians’ perception of sCr clinical significance perioperatively. Finally, the incidence and risk factors associated with PO-AKI and renal function deterioration within a year postoperatively will also be evaluated.

## Methods

### Study design

In this single-centre, observational retrospective study, we included all patients undergoing major elective general abdominal surgery from November 2016 to February 2017 at the Department of Anaesthesia of Careggi Hospital, Florence, Italy. The Institutional Review Board of Area Vasta Toscana Centro approved this study with the reference number CEAVC Reg N. 13881. Because of the retrospective nature of this study, written consent for analysis and publication of clinical data was waived by the IRB.

As a primary endpoint, a flow chart will describe (1) the number of patients actually screened for PO-AKI, (2) the incidence of PO-AKI in those patients for whom sCr was available postoperatively, (3) the number of patients actually screened for long-term reduction of kidney function (both those who presented PO-AKI and those who did not), and (4) the incidence of long-term kidney impairment. Furthermore, a quantitative survey will explore local physicians’ perspectives on the clinical utility of sCr in identifying patients at high risk to develop AKI.

### Patients selection criteria

We collected data from patients older than 18 years who underwent major oncologic surgery under general anaesthesia and who had sCr measured preoperatively. Patients who underwent chemotherapy were excluded from the analysis (mainly because they are usually screened with a protocolised lab evaluation before chemotherapy infusion), as well as those who died before 12-month follow-up. Based on data available in medical and surgical records, preoperative, intraoperative, and postoperative variables were retrospectively considered for each patient. In particular, patients’ demographics (i.e. age, sex, preoperative risk factors for AKI, preoperative values of sCr, and the corresponding eGFR according to the CKD-EPI formula (Sharma et al. 2014)) and intraoperative data (including the type of surgery and surgical technique) were recorded for each case. Postoperative in-hospital complications (i.e. postoperative sepsis, shock, or respiratory failure); sCr values on the first, second, and third postoperative day; and in-hospital mortality were also recorded. Glycopeptides, aminoglycoside, iodinated contrast media, and non-steroidal anti-inflammatory drugs were considered as nephrotoxic drugs. Metformin was already routinely discontinued prior to surgery, according to local standards of care. For each patient, electronic medical records of the first year postoperatively were analysed.

### PO-AKI assessment

For each patient, the presence of PO-AKI was retrospectively assessed within the first 3 days postoperatively using the KDIGO criteria (Kellum et al. 2012). Specifically, PO-AKI was defined as an increase in sCr by ≥ 0.3 mg/dl within 48 h or at least ≥ 1.5 times the baseline preoperative value. Due to the lack of data on urinary output, only sCr was used to determine the incidence and severity of AKI. Patients were thus retrospectively divided into two groups: AKI and No-AKI. Preoperative and intraoperative factors associated with PO-AKI were identified through multivariate logistic regression analysis.

### LT-KDys assessment

The sCr value measured at month 12 postoperatively was used to identify long-term reduction of kidney function within 1 year after surgery. In particular, long-term kidney dysfunction (LT-KDys) was defined as a decrease in eGFR of at least 10 ml/min/1.73 m^2^ at 1 year compared to the baseline (preoperative). Patients were divided into those who had a long-term progression of the kidney dysfunction (LT-KDys group) and those who had not (No-LT-KDys group). Preoperative, intraoperative, and postoperative factors associated with LT-KDys were identified through multivariate logistic regression analysis.

### Quantitative survey

A questionnaire was administered to 125 physicians to collect information on local attitudes regarding the use of sCr perioperatively and its relationship with PO-AKI. All participants were engaged in the care of patients enrolled in this study. Participants were grouped into three groups: anaesthesiologists (24%), surgeons (24%), and residents/fellows (in anaesthesiology 26% and in general surgery 26%). The survey is divided into two sections: the first section collected personal data, including participant gender and age; the second section encompasses 10 statements based on literature data (Digvijay et al. 2019; Kellum et al. 2012; Romagnoli et al. 2016) with a scale of answer options ranging from 0 (disagree) to 10 (strongly agree). For each statement, we calculated the median scores and interquartile range [IQR].

### Statistical analysis

Incidences of PO-AKI and LT-KDys are expressed in percentage form. Continuous variables are presented as means ± standard deviations or median and interquartile range, according to the data distribution (normality assessed through the Shapiro-Wilk test). Categorical variables are expressed as percentages. Both for PO-AKI and LT-KDys, a multivariate logistic regression model was designed considering the quantitative and qualitative variables associated with the event at univariate analysis with a *p* value < 0.15. A stepwise selection procedure based on the Akaike Information Criterion (AIC) was used to select the variables in the final model. The results are expressed in terms of *p* value, odds ratio (OR), and 95% confidence interval (95%CI). The analysis was performed using the R© software version 3.5.1.

## Results

**Fig. 1:**
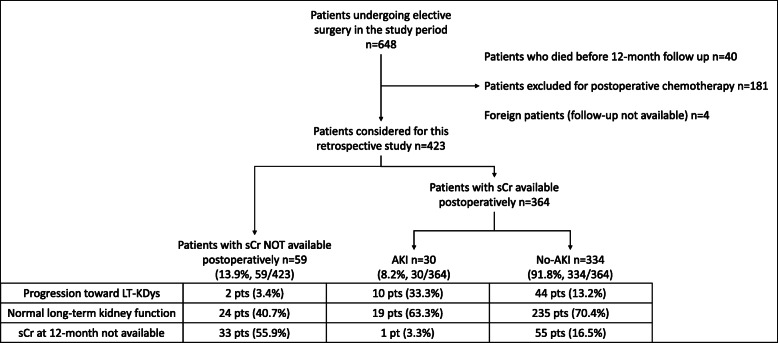
Patient selection, PO-AKI, and long-term outcomes

Six hundred and forty-eight patients underwent major abdominal surgery in the study period (Fig. 1). Among them, 40 were excluded because they died before 12-month follow-up; in this group of patients, 17 (17/40, 42.5%) had PO-AKI. Furthermore, 181 patients were excluded due to postoperative chemotherapy, while four patients were excluded because they were foreigners (follow-up data were unavailable). Four hundred and twenty-three patients were thus considered for this study. Of these, only 364 (86.1%) had at least one sCr value available during the first three postoperative days (Fig. 1). In this group, PO-AKI was detected in 30 patients (30/364, 8.2%, see Fig. 1). Among them, 28 had AKI stage 1 while 2 had AKI stage 2. The long-term kidney function of patients is described in Fig. 1.

Patients in the AKI group were older; predominantly male; presented with pre-existing vascular disease, higher baseline sCr, and mainly CKD; were often treated with nephrotoxic drugs preoperatively; and generally had more preoperative risk factors for PO-AKI (univariate analysis in Table 1).

**Table 1:** Preoperative and intraoperative factors for patients who had a postoperative evaluation of serum creatinine available for PO-AKI detection (PO-SCr-available) and for patients who did not (SCr result not available). In those patients for whom postoperative serum creatinine was measured, preoperative and intraoperative variables statistically associated with PO-AKI have been evaluated through univariate analysis. For qualitative data with more than two levels (e.g. type of surgery), the Wald test was used to provide an overall *p* value. For the numbers of preoperative risk factors for AKI, *n*<2 is used as a reference for the other levels, and videolaparoscopic is used for the other surgical approaches. The results are presented as OR [95%CI] and *p* value

	SCr result not available (*n*=59)	PO-SCr-available				
		Total (*n*=364)	No-AKI (*n*=334)	AKI (*n*=30)	OR	*p*
Male gender	32 (54.2%)	186 (51.1%)	164 (49.1%)	22 (73.3%)	2.85 [1.23; 6.58]	0.013
Age (yrs)	67.6 [52.4–76.1]	70.2 [60.5–78.1]	70.1 [60.1–77.7]	77.53 [68.9–82.2]	1.03 [1.00; 1.07]	0.018
Baseline SCr (mg/dl)	0.83 [0.66–1.02]	0.83 [0.71–0.99]	0.81 [0.68–0.97]	1.06 [0.82–1.38]	2.55 [1.31; 4.96]	<0.001
Baseline eGFR (ml/min/1.73 m^2^)	81.9 [73.2–97.4]	81.4 [68.1–93.5]	81.9 [69.1–93.9]	67.6 [45.1–82.9]	0.97 [0.95; 0.98]	<0.001
Comorbidities						
Diabetes	3 (5.1%)	56 (15.4%)	49 (14.7%)	7 (23.3%)	1.77 [0.72; 4.35]	0.196
Hypertension	22 (37.3%)	181 (49.7%)	162 (48.5%)	19 (63.3%)	1.83 [0.85; 3.97]	0.131
Vascular disease	5 (8.5%)	47 (12.9%)	39 (11.7%)	8 (26.7%)	2.75 [1.15; 6.6]	0.040
Previous solid neoplasm	40 (67.8%)	231 (63.5%)	213 (63.7%)	18 (60%)	0.75 [0.28; 2.04]	0.814
Previous haematologic neoplasm	1 (1.7%)	6 (1.7%)	6 (1.8%)	0	–	–
Chronic heart failure	2 (3.4%)	50 (13.7%)	43 (12.9%)	7 (23.3%)	2.06 [0.83; 5.09]	0.159
Preop nephrotoxic drugs	16 (27.1%)	144 (39.6%)	126 (37.7%)	18 (60%)	2.48 [1.15; 5.31]	0.020
CKD	4 (6.8%)	61 (16.8%)	47 (14.1%)	14 (46.7%)	5.34 [2.45; 11.7]	<0.001
Number of preop risk factors for AKI						0.010
<2	38 (64.4%)	172 (47.3%)	174 (52.1%)	7 (23.3%)	ref.	
>2	13 (22.0%)	74 (20.3%)	66 (19.8%)	8 (26.7%)	2.76 [0.80–9.54]	0.109
>3	5 (8.5%)	65 (17.9%)	59 (17.7%)	6 (20.0%)	2.31 [0.63–8.55]	0.208
>4	3 (5.1%)	30 (8.2%)	26 (7.8%)	4 (13.3%)	3.50 [0.82–15.0]	0.091
>5	0	13 (3.6%)	8 (2.4%)	5 (16.7%)	14.20 [3.17–63.7]	<0.001
>6	0	1 (0.3%)	1 (0.3%)	0	–	–
Type of surgery						
Colorectal	38 (64.4%)	255 (70.1%)	235 (70.4%)	20 (66.7%)	1.25 [0.52; 3.01]	0.831
Gastric	9 (15.3%)	31 (8.5%)	29 (8.7%)	2 (6.7%)	0.67 [0.15; 2.96]	1.000
Oesophageal	0	9 (2.5%)	8 (2.4%)	1 (3.3%)	1.41 [0.17; 11.6]	0.543
Hepatic	1 (1.7%)	16 (4.4%)	16 (4.5%)	0	–	–
Pancreatic	3 (5.1%)	19 (5.2%)	17 (5.1%)	2 (6.7%)	1.33 [0.29; 6.06]	0.663
Others	8 (13.6%)	31 (8.5%)	26 (7.8%)	5 (16.7%)	3.34 [1.43; 7.82]	0.08
Surgical approach						0.67
Videolaparoscopy	27 (45.7%)	134 (36.8%)	125 (37.4%)	9 (30%)	ref.	
Laparotomy	27 (45.7%)	201 (55.2%)	183 (54.8%)	18 (60%)	1.36 [0.59; 3.13]	0.46
Robotic surgery	5 (8.5%)	29 (8%)	25 (7.5%)	4 (13.3%)	1.72 [0.43; 6.83]	0.44

*Abbreviations*: *CKD*, chronic kidney disease; *SCr*, serum creatinine; *yrs*, years

According to results from multivariate logistic regression analysis, preoperative and intraoperative factors statistically associated with PO-AKI were male gender (OR 2.62 [1.11; 6.21], *p*=0,003), pre-existing CKD (OR 5.61 [2.54; 12.4], *p*<0.001), and perioperative use of nephrotoxic drugs (OR 2.32 [1.05; 5.10], *p*=0.04). Interestingly, one patient (3.3%) in the AKI group was not evaluated long term after surgery.

Among the 59 patients (13.9% of the 423 taken into consideration) in whom PO-AKI was not assessable, 4 (6.8%) had pre-existing CKD, 5 (8.5%) had more than three risk factors preoperatively for perioperative AKI, and 3 (5.1%) had more than four risk factors (Table 1). In 33 out of the 59 patients (55.9%), sCr results were not available even during the 1-year follow-up after surgery (Fig. 1). During the follow-up period, sCr at 12 months was available for 334 patients. In 89 out of 423 patients (21.04%), long-term evaluation of kidney function was missing (Table 2).

**Table 2:** Preoperative, intraoperative, and postoperative factors among patients who had 12-month follow-up evaluation of serum creatinine available for LT-KDys detection (FU-SCr-available) and among patients who did not (SCr result not available). Among patients who had long-term serum creatinine, preoperative, intraoperative, and postoperative variables statistically associated with LT-KDys have been evaluated through univariate analysis. For qualitative data with more than two levels (e.g. type of surgery), the Wald test is used to provide an overall *p* value. For the numbers of preoperative risk factors for AKI, *n*=1 is used as a reference for the other levels, as well as the videolaparoscopic for the other surgical approach. The results presented as OR [95%CI] and *p* value

	SCr result not available (*n* = 89)	FU-SCr-available				
		Total (*n* = 334)	No-LT-KDys (*n* = 278)	LT-KDys (*n* = 56)	OR	*p*
Male gender	43 (48.3%)	175 (52.4%)	144 (51.8%)	31 (55.4%)	1.15 [0.65; 2.05]	0.662
Age (yrs)	67.3 [53.7–75.6]	70.3 [60.8–78.2]	70.3 [61.0–78.1]	69.9 [56.5–78.6]	0.99 [0.97; 1.01]	0.475
Baseline eGFR(ml/min/1.73 m^2^)	81.5 [72.0–94.6]	81.3 [68.1–72.0]	80.7 [68.1–92.3]	83.3 [68.9–102.2]	1.02 [1; 1.03]	0.049
Baseline sCr (mg/dl)	0.81 [0.72–1.02]	0.83 [0.68–1.00]	0.83 [0.71–1.00]	0.76 [0.65–0.98]	0.62 [0.26; 1.48]	0.127
Comorbidities						
Diabetes	10 (16.9%)	49 (83.1%)	40 (81.6%)	9 (18.4%)	1.14 [0.52; 2.51]	0.685
Hypertension	34 (16.7%)	169 (83.3%)	141 (83.4%)	28 (16.6%)	0.97 [0.55; 1.73]	1.000
Vascular disease	4 (7.7%)	48 (92.3%)	40 (83.3%)	8 (16.7%)	0.99 [0.44; 2.25]	1.000
Previous solid neoplasm	52 (19.2%)	219 (80.8%)	183 (83.6%)	36 (16.4%)	1.35 [0.69; 2.65]	0.371
Previous hematologicneoplasm	2 (28.6%)	5 (71.4%)	5 (100%)	0	–	–
Chronic heart failure	7 (13.5%)	45 (86.5%)	34 (75.6%)	11 (24.4%)	1.75 [0.83; 3.72]	0.139
Nephrotoxic drugs	28 (17.5%)	132 (82.5%)	108 (81.8%)	24 (18.2%)	1.18 [0.66; 2.11]	0.653
CKD	13 (20%)	52 (80%)	42 (80.8%)	10 (19.2%)	1.22 [0.57; 2.61]	
Numbers of preop. riskfactors for AKI						0.416
>2	16 (17.9%)	71 (21.3%)	63 (22.7%)	8 (14.3%)	0.69 [0.27; 1.78]	0.447
>3	13 (14.6%)	57 (17.1%)	49 (17.6%)	8 (14.3%)	0.89 [0.34; 2.31]	0.814
>4	6 (6.7%)	27 (8.1%)	20 (7.2%)	7 (12.5%)	1.91 [0.67; 5.43]	0.224
>5	1 (1.1%)	12 (3.6%)	8 (2.9%)	4 (7.1%)	2.73 [0.71; 10.4]	0.141
>6	0	1 (0.3%)	1 (0.4%)	0	–	–
Type of surgery						
Colorectal	59 (20.1%)	234 (79.9%)	192 (82.1%)	42 (17.9%)	1.24 [0.63; 2.44]	0.620
Gastric	6 (15%)	34 (85%)	30 (88.2%)	4 (11.8%)	0.57 [0.19; 1.68]	0.361
Oesophageal	0	9 (100%)	8 (88.9%)	1 (11.1%)	0.7 [0.08; 5.84]	1.000
Hepatic	3 (17.6%)	14 (82.4%)	12 (85.7%)	2 (14.3%)	0.82 [0.18; 3.77]	1.000
Pancreatic	4 (18.2%)	18 (81.8%)	17 (94.4%)	1 (5.6%)	0.28 [0.03; 2.14]	0.328
Others	15 (38.5%)	24 (61.5%)	19 (79.2%)	5 (20.8%)	1.87 [0.85; 4.09]	0.116
PO complications	5 (7.2%)	64 (92.8%)	50 (70.1%)	14 (21.9%)	1.52 [0.77; 2.99]	0.263
Cardiogenic Shock	2 (12.5%)	14 (87.5%)	7 (50%)	7 (50%)	5.53 [1.86; 16.5]	0.030
Septic shock	1 (2.4%)	40 (97.6%)	33 (82.5%)	7 (17.5%)	1.06 [0.44; 2.53]	0.825
Haemorrhagic shock	2 (10%)	18 (90%)	15 (83.3%)	3 (16.7%)	0.99 [0.28; 3.55]	1.000
Stroke	0	2 (100%)	2 (100%)	0	–	–
SCr 24 h vs. baseline SCr	−0.08 [−0.20 to −0.08]	−0.01 [−0.10–0.12]	−0.02 [−0.11 to −0.09]	0.08 [−0.04–0.15]	2.14 [0.98; 4.69]	0.03
SCr 48 h vs. baseline SCr	−0.09 [−0.17–0.01]	−0.01 [−0.16–0.06]	−0.07 [−0.18–0.04]	0.04 [−0.05–0.20]	3.04 [1.24; 7.46]	<0.001
PO-AKI	1 (1.1%)	29 (8.7%)	19 (6.8%)	10 (17.9%)	2.96 [1.30; 6.78]	0.010

*Abbreviations*: *CKD*, chronic kidney disease; *SCr*, serum creatinine; *yrs*, years

Among the 334 patients, 56 (16.8%) developed LT-KDys; these patients had a lower preoperative eGFR and more frequently developed postoperative shock and PO-AKI.

Multivariate logistic regression analysis showed that preoperative cardiovascular comorbidities (OR 2.92 [1.19; 7.15], *p*=0.02), perioperative shock (OR 3.44 [0.97; 12.10], *p*=0.05), and PO-AKI (OR 3.87 [1.46; 10.30], *p*=0.01) were independently associated with LT-KDys. In addition, patients in the LT-KDys group had higher increases in postoperative sCr than those in the No-LT-KDys group (difference between sCr at 24h and baseline sCr, 0.08 vs. − 0.02, *p*=0.003). Among the remaining 89 patients without a sCr result available in the 12-month follow-up period, 1 (1.1%) had PO-AKI.

A total of 83 participants responded to the questionnaire, yielding a response rate of 66.4%. Participants were equally distributed among men and women, and their median age was 51 years (range, 32–63) for anaesthesiologists and surgeons and 29 years (range, 24–33) for fellows and residents. The results of the survey are summarised in Table 3.

**Table 3:** Qualitative survey exploring the local attitudes regarding the use of sCr perioperatively and its relationship with PO-AKI

	Anaesthesiologists, *n*=22 (26.5%)	Surgeons, *n*=15 (18.1%)	Fellows/residents, *n*=46 (55.4%)
1 Preoperative measurement of sCr is required for perioperative risk stratification.	10 [9–10]	10 [9–10]	10 [9–10]
2. Preoperative measurement of sCr is required to define a perioperative nephroprotective strategy for high-risk patients.	9 [8–10]	8 [8–9]	9 [9–10]
3. Preoperative measurement of sCr is required to define a perioperative anaesthesiological and surgical strategy (e.g. choice of anaesthetics drugs, surgical approach).	10 [9–10]	7 [6–8]	8 [7–10]
4. Preoperative measurement of sCr might help to identify those patients at high risk to develop AKI and for whom a serial postoperative sCr assessment is needed.	10 [9–10]	9 [8–10]	10 [9–10]
5. Postoperative measurement of sCr is always required after major oncological abdominal surgery.	10 [9–10]	9 [8–10]	9 [9–10]
6. Postoperative measurement of sCr should be more systematic in those patients at high risk to develop AKI.	10 [9–10]	9 [8–10]	9 [9–10]
7. Doubling of postoperative sCr from the baseline preoperative value identifies AKI and is a severe condition that affects the short- and long-term prognosis of patients.	10 [9–10]	9 [9–10]	9 [9–10]
8. An increase in postoperative sCr of 0.3 mg/dl from the baseline preoperative value identifies AKI and is a severe condition that affects the short- and long-term prognosis of patients.	9 [9–10]	7 [5–9]	7 [6–9]
9. Postoperative AKI is a dangerous condition that might affect the patient’s long-term kidney function leading to CKD or ESRD.	10 [9–10]	9 [7–10]	9 [7–9]
10. In patients with postoperative AKI, long-term follow-up evaluation of renal function and specialist nephrology referral might be required.	9 [8–10]	8 [8–10]	8 [8–10]

The 10-item questionnaire showing the average scores and IQR in each group

*Abbreviations*: *sCr*, serum creatinine; *AKI*, acute kidney injury; *CKD*, chronic kidney disease; *ESRD*, end-stage renal disease

## Discussion

In this retrospective observational study, we found that only 86.1% of oncological patients undergoing major abdominal surgery in a tertiary university hospital had at least one sCr value available to detect postoperative kidney impairment. Among these, PO-AKI was present in 8.2% of cases. We do not have data on long-term kidney function for 21% of the patients enrolled, despite the fact that PO-AKI was detected in 3.3% of them. Interestingly, 33 of 423 patients (7.8%) had no sCr immediately postoperatively or in the long-term follow-up period, although all the physicians engaged with the care of those patients recognised that postoperative assessment of sCr is required after major oncological abdominal surgery, particularly in patients at high risk to develop PO-AKI and LT-KDys (see Table 3).

PO-AKI is a very well-known postoperative complication occurring in patients undergoing cardiac and non-cardiac surgery (Romagnoli and Ricci 2015). Wang and Bellomo showed that the incidence of cardiac surgery-associated AKI ranged from 5 to 42% (Wang and Bellomo 2017); in comparison, Long et al. showed that AKI after major abdominal surgery occurred in 12 to 22% of the patients (Long et al. 2016). This wide variability might be related to the differences in case mix and diagnostic criteria used to define AKI. In a prospective study, we previously showed a 12% incidence of PO-AKI in a cohort of adults undergoing major abdominal surgery (Romagnoli et al. 2016). A slightly smaller incidence of PO-AKI (8.2%) has been observed in the present study, notwithstanding the similarity of the cohorts observed. The lack of urinary output criteria in this retrospective study might explain the slightly smaller incidence of PO-AKI compared to that reported in the previous prospective study (where AKI was defined using both serum creatinine and urinary output criteria of AKIN classification).

There may be a widespread lack of awareness of the clinical relevance of PO-AKI in clinical practice. Indeed, while several technologically advanced tools and/or strategies for early identification of high-risk patients and PO-AKI are currently developed and described in the literature, sCr still remains the most used biomarker. In our centre, newly developed biomarkers, technologically advanced sniffers, or minimally invasive renal stress tests are not routinely available in clinical practice. Although physicians theoretically appreciate the diagnostic and prognostic role of perioperative sCr, in practice, they seem to miss the opportunity to measure sCr and individualise patients’ clinical management. Being a retrospective study, the exact reasons for not measuring sCr after major surgery remain not clear although inadequate perioperative and post-discharge communication among anaesthesiologists, surgeons, and nephrologists may be hypothesised. Of note, in patients in whom sCr was measured postoperatively, a proportion of patients with PO-AKI did not have a repeat sCr measurement either before hospital discharge or at 1-year follow-up or a referral for nephrology follow-up.

Our analysis identified similar risk factors for PO-AKI as reported in the literature: preoperative CKD, older age, and perioperative use of nephrotoxic drugs (An et al. 2018). Ideally, these risk factors should prompt clinicians to intensify perioperative monitoring for early diagnosis of PO-AKI. In particular, the early recognition of high-risk patients provides an opportunity for the adoption of diagnostic and therapeutic measures to prevent renal impairment such as goal-directed fluid therapy based on physiological parameters in order to optimise oxygen delivery, avoidance of drugs that can impair renal function, and strict glycaemic control.

In this retrospective study, 59 patients (13.9%) had no sCr assessed postoperatively, despite the fact that they had undergone major abdominal surgical procedures for a malignant disease. Interestingly, 4 of them (6.8%) had pre-existing CKD, 5 (8.5%) had more than three preoperative risk factors for perioperative AKI, and 3 (5.1%) had more than four (Table 1). Thus, all of them were potential high-risk patients. Yet, serial assessment of postoperative sCr was not undertaken, and opportunities for intervention were missed. Furthermore, not all patients with PO-AKI received perioperative surveillance, specific recommendations, or specialised nephrology consultations, and more importantly, for some of them (3.3%), long-term renal function was not assessed at all.

Our results show that preoperative cardiovascular comorbidities, perioperative shock, and PO-AKI were significantly associated with LT-KDys. The identification of these risk factors should prompt clinicians to intensify postoperative long-term monitoring for early diagnosis of CKD. Notably, among patients without a sCr available postoperatively, 33 (55.9%) did not have a renal long-term follow-up after surgery (Fig. 1). For these patients, renal prognosis is unknown.

We recognise that our study has some limitations. First, this is a single-centre retrospective analysis, and some sCr results were missing which impacts the incidence of PO-AKI. Second, we only used sCr results to diagnose AKI and acknowledge that the incidence of PO-AKI might be underestimated. Third, data on fluid administration and fluid balance were not available to us. We acknowledge that sCr concentration is influenced by fluid accumulation (De Rosa et al. 2016). Fourth, all patients enrolled in this study underwent surgery for malignant disease. Although we excluded patients who underwent postoperative chemotherapy, we recognise that patients were exposed to other nephrotoxic agents in the first year postoperatively. Thus, the development of LT-kDys might be caused by more factors than PO-AKI alone. Fifth, we were unable to reliably collect data on intraoperative blood loss, hypotension, blood transfusion, total vasopressor use, diuretic administration, and duration of surgery (Wilson et al. 2016). Finally, we were not able to fully investigate the exact reasons for not measuring sCr after major surgery in our centre due to the retrospective nature of the study design. Thus, the results presented in this study should be taken as preliminary findings, which we believe warrant external validation through a prospective multicentre study.

## Conclusion

In this single-centre, retrospective observational study, we showed that, despite the frequent occurrence of PO-AKI in oncological adult patients undergoing elective major abdominal surgery, sCr was not measured systematically in the postoperative period, even when risk factors for AKI were known preoperatively. Similarly, in cases where a significant decline in GFR was observed at 12 months after surgery, long-term follow-up of kidney function was rarely performed, even in patients at high risk for LT-KDys.

## Data Availability

The datasets analysed during the current study are available from the corresponding author on reasonable request.

## Abbreviations

AIC: Akaike Information Criterion
AKI: Acute kidney injury
CKD: Chronic kidney disease
FU-SCr-available: Follow-up evaluation of serum creatinine available for LT-KDys detection
LT-KDys: Long-term kidney dysfunction
OR: Odds ratio
PO-AKI: Postoperative acute kidney injury
PO-SCr-available: Postoperative evaluation of serum creatinine available for PO-AKI detection
RFR: Renal functional reserve
sCr: Serum creatinine
